# Measuring nestedness: A comparative study of the performance of different metrics

**DOI:** 10.1002/ece3.6663

**Published:** 2020-10-22

**Authors:** Clàudia Payrató‐Borràs, Laura Hernández, Yamir Moreno

**Affiliations:** ^1^ Laboratoire de Physique Théorique et Modélisation UMR08089 CNRS‐CY Cergy‐Paris University Cergy‐Pontoise Cedex France; ^2^ Institute for Biocomputation and Physics of Complex Systems (BIFI) University of Zaragoza Zaragoza Spain; ^3^ Department of Theoretical Physics, Faculty of Sciences University of Zaragoza Zaragoza Spain; ^4^ ISI Foundation Turin Italy

**Keywords:** ecological networks, maximum Entropy, nestedness, null model

## Abstract

Nestedness is a property of interaction networks widely observed in natural mutualistic communities, among other systems. A perfectly nested network is characterized by the peculiarity that the interactions of any node form a subset of the interactions of all nodes with higher degree. Despite a widespread interest on this pattern, no general consensus exists on how to measure it. Instead, several nestedness metrics, based on different but not necessarily independent properties of the networks, coexist in the literature, blurring the comparison between ecosystems. In this work, we present a detailed critical study of the behavior of six nestedness metrics and the variants of two of them. In order to evaluate their performance, we compare the obtained values of the nestedness of a large set of real networks among them and against a maximum‐entropy and maximum‐likelihood null model. We also analyze the dependencies of each metrics on different network parameters, as size, fill, and eccentricity. Our results point out, first, that the metrics do not rank networks universally in terms of their degree of nestedness. Furthermore, several metrics show significant dependencies on the network properties considered. The study of these dependencies allows us to understand some of the observed systematic shifts against the null model. Altogether, this paper intends to provide readers with a critical guide on how to measure nestedness patterns, by explaining the functioning of several metrics and disclosing their qualities and flaws. Besides, we also aim to extend the application of null models based on maximum entropy to the scarcely explored area of ecological networks. Finally, we provide a fully documented repository that allows constructing the null model and calculating the studied nestedness indexes. In addition, it provides the probability matrices to build the null model for a large dataset of more than 200 bipartite networks.

## Graphical abstract

Several metrics aiming at quantifying nestedness, based on different but not necessarily independent properties of the networks, coexist in the literature blurring the comparison between ecosystems. In this work, we present a detailed critical study of the behavior of various nestedness metrics, analyzing its dependencies, advantages, and flaws.

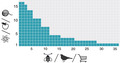

## 1 INTRODUCTION

The characterization of mutualistic networks has been the ground of considerable debate during the last decades. This type of network is represented as a graph that codifies mutually beneficial interactions, namely the species of the network involved in these interactions naturally obtain a benefit from them, even if the nature of the benefits might be different. This is the case, for instance, of plant–pollinator communities where pollinators feed on flower's nectar while plants assure their reproduction. Moreover, as mutualistic interactions often take place only between species of different kinds, they can therefore be represented by a *bipartite network*, characterized by two disjoint sets of vertices (or nodes) representing the species, with the edges (or links) joining only vertices of different kinds, that is, links connect species of the two branches of the bipartite graph.

The structure and dynamics of mutualistic networks have received increasing attention due, in particular, to the role that mutualism is assumed to play in the complexity–stability paradox as a stabilizer of large and complex communities (McCann, 2000). Indeed, it has generally been admitted that mutualistic interactions enhance stability by screening competition (Bastolla et al., 2009; Thébault & Fontaine, 2010), though this idea has recently been challenged (Gracia‐Lázaro, Hernández, Borge‐Holthoefer, & Moreno, 2018; James, Pitchford, & Plank, 2012; Pascual‐García & Bastolla, 2017). Furthermore, the observation of natural ecosystems has revealed that in a vast majority of cases, mutualistic interactions are not uniformly distributed. Instead, the species interact in a very particular way, leading to a network structure called *nestedness* (Bascompte, Jordano, Melián, & Olesen, 2003).

**Figure 1:**
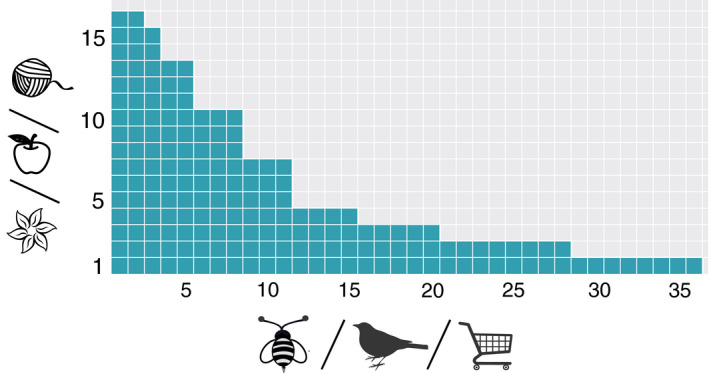
Scheme of a perfectly nested bipartite matrix. The region in green represents the “1s” in the biadjacency matrix, and the one in light gray the “0s”. Nodes are ordered by increasing ranking (decreasing degree) from bottom to top and from left to right. Since the network is perfectly nested, the interactions of a given node are always a subset of the interactions of the nodes with smaller ranking. We portray three different possible types of paradigmatic mutualistic networks: flowering plants and its pollinators, fruit‐producing plants and its seed‐disperser birds, and sellers, and buyers. The figure also shows how different nodes may have the same degree, leading to some degeneracy in the ordering

**Figure 2:**
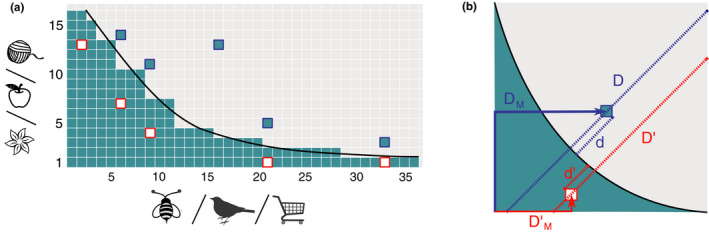
Continuous approximation of a maximally packed nested matrix. (a) Scheme of a nonperfectly nested network. Green squares depict the interactions, and the black curve represents the *Isocline of perfect nestedness* (IPN). The unexpected interactions above the IPN are highlighted in blue, while absent interactions below the IPN are highlighted in red. (b) The mapping of a matrix into the unit square. The black curve corresponds to the IPN, and unexpectedly present (absent) interactions are highlighted in blue (red). The figure shows two different kinds of distances that may be used for measuring nestedness: *D*, *D*′, *d,* and *d*′ (traced in dashed lines) are used in the calculation of the temperature, while *D*_M_ and $D_{M}^{\prime }$ (represented by solid arrows) are used by the nestedness index based on Manhattan distance (NMD)

A network is said to be *perfectly nested* when the contacts of a species of a given degree are a subset of the contacts of all the species of larger degree, as illustrated in Figure 1. The system is then composed of generalist and specialist species in each guild, the former interacting with a large amount of the possible counterparts and the latter only with generalists, in such a way that specialist–specialist interactions are mostly absent (Bascompte et al., 2003). As a consequence, when the nodes of one guild are ordered by decreasing (or increasing) degree, the nodes of the other guild appear automatically ordered in the same way, and the corresponding biadjacency matrix has all its nonzero elements on the same side of a curve called “isocline of perfect nestedness” (IPN) (Atmar & Patterson, 1993), see Figure 2. This ordering leads to the characteristic triangular shape (Medan et al., 2007) shown in Figure 1. The arrangement of the matrix which reveals and maximizes its nestedness is usually referred to as a *maximally packed configuration*, and various methods to produce it can be found in the literature (Domínguez‐García & Munoz, 2015; Lin, Tessone, & Mariani, 2018; Rodríguez‐Gironés & Santamaría, 2006).

Although natural mutualistic ecosystems are not perfectly nested, the same general pattern with some fluctuations is found in a large variety of known ecosystems that correspond to a wide range of different geographic and climatic conditions and involve very different species (Bascompte et al., 2003; Guimaraes, Rico‐Gray, Furtado dos Reis, & Thompson, 2006; Kondoh, Kato, & Sakato, 2010; Vázquez, Poulin, Krasnov, & Shenbrot, 2005). Despite that nested patterns have mainly been observed and studied in ecology, systems displaying nested forms of interactions have also been reported for a variety of economic and social networks. For instance, nestedness has been detected as well in trading relationships between countries (De Benedictis & Tajoli, 2011; König, Tessone, & Zenou, 2014) and individuals (Hernández, Vignes, & Saba, 2018), in manufacturer–contractor networks (Saavedra, Reed‐Tsochas, & Uzzi, 2009), communication systems (Borge‐Holthoefer, Baños, Gracia‐Lázaro, & Moreno, 2017), cultural assemblages (Kamilar & Atkinson, 2014), and even scientific production (Cimini, Gabrielli, & Labini, 2014).

The astounding ubiquity of nestedness in natural systems called for the need of, on the one hand, having a good indicator to quantify it, and at the same time, providing answers to challenging questions about the reasons behind such a generally observed pattern and its consequences. Regarding the latter problem, a recent work (Payrató‐Borràs, Hernández, & Moreno, 2019) has shown analytically and numerically that nestedness is not an irreducible global property, but that it emerges instead from local properties of the network. In particular, it arises as an entropic consequence of the double heterogeneity observed in the degree sequences of natural ecosystems. Indeed, by building a maximum‐entropy ensemble with the constraint that the observed degree sequence is kept on average, such that this sequence is found with maximum probability in the ensemble, one finds that nestedness values of real networks do not significantly differ from the average of the random ensemble. Ulrich and Gotelli (2007) and Jonhson, Domínguez‐García, and Muñoz (2013) had also observed this fact when randomizing real networks but fixing the degree sequences exactly (the so‐called FF model, see below). However, the poor statistics that results from such strong constraint limited the understanding of the extent of their observations. In any case, these different works point out that the real degree sequences provide sufficient structural information to reproduce the observed nestedness. Besides, in what concerns the dynamical explanation of the origin of the pattern, some articles have recently shown that it may emerge as the biproduct of an assembly process that does not explicitly seek to generate nestedness (Maynard, Serván, & Allesina, 2018; Valverde et al., 2018). Altogether, these structural and dynamical arguments support the notion of nestedness as an architectural spandrel determined by the degree sequences, rather than a significant, evolutionary selected pattern in its own.

All in all, the fact that nestedness is not an independent emergent pattern does not invalidate its usefulness. For instance, its global character makes it a helpful tool to detect heterogeneity in species' connectivity, by using a single parameter. Given that the observed degree distributions are highly heterogeneous, it is tempting to characterize them by the exponent of a power‐law fit, and several attempts were made in this sense (Jordano, Bascompte, & Olesen, 2003). However, the typical small size of natural ecosystems makes such fit meaningless. Therefore, nestedness continues to be a useful and widely studied global pattern, and the aforementioned question of how to measure it remains an essential challenge.

Admittedly, as a result of the effort to quantify nestedness, a variety of *metrics* with their corresponding *nestedness indices* coexist in the ecological literature. However, since they are based on diverse but not necessarily independent properties of the nested networks, the comparison of the degree of nestedness of different ecosystems remains unclear. The situation recalls the well‐known history of the definition of temperature in Thermodynamics. Initially defined operationally, that is, by listing the protocol to measure it, the obtained temperature values suffered from the flaw that they depended on the thermometer used. This problem was solved by the theoretical definition of the temperature based on the Second Principle of Clausius, and finally, the notion of temperature was completely understood by the microscopic approach of Statistical Physics introduced by Boltzmann and Gibbs. Interestingly, the first metrics of nestedness defined by Atmar and Patterson was called *temperature* (Atmar & Patterson, 1993). This initial proposal was followed by a long struggle to find the best index to measure nestedness, with the development of various approaches ranging from algorithmic procedures to analytical methods. A review of the early nestedness indices by Ulrich and Gotelli was published in 2009 (Ulrich, Almeida‐Neto, & Gotelli, 2009), while a more recent review by Mariani, Ren, Bascompte, and Tessone (2019) provides a very detailed and updated summary of the most common nestedness metrics.

Nonetheless, the metrics defined to quantify nestedness suffer from a critical drawback: As they are strongly dependent on different network parameters (like size and fill), the comparison among ecosystems is difficult, even in the case where the same metrics (the same thermometer) is used to measure all the systems. Some of these problems have been reported by several authors, notably on the occasion of the introduction of each new index and/or package devoted to correct some of the shortcomings of previously existing ones (Almeida‐Neto, Guimarães, Guimarães, Loyola, & Ulrich, 2008; Burgos, Ceva, Hernández, & Perazzo, 2009; Dormann, Fründ, Blüthgen, & Gruber, 2009; Grimm & Tessone, 2017; Rodríguez‐Gironés & Santamaría, 2006; Staniczenko, Kopp, & Allesina, 2013; Ulrich et al., 2009). Still, these works mainly focus on the dependence on the size and the density of links of the network of a few metrics, leaving aside other important nestedness indices—either more recent or less popular—as well as the interdependencies among network parameters.

In order to overcome the aforementioned difficulties when measuring and comparing the nestedness of different networks, the standard procedure is to contrast the nestedness value of a given real network with that of a *null model*, both calculated using the same metrics. A null model is an ensemble of networks obtained by the randomization of the natural system under study, imposing some constraints. Different constraints lead, then, to different null models of the same real network (Payrató‐Borràs et al., 2019; Ulrich & Gotelli, 2007). In a majority of cases, such constraints are enforced algorithmically. In particular, a very popular choice is the *fixed‐fixed* null model (FF herein), where the degree sequences are strictly kept and null networks are produced through numerical randomizing procedures (Gotelli & Entsminger, 2001). On the other hand, the family of null models based on maximum‐entropy ensembles precludes the algorithmic randomization (Squartini & Garlaschelli, 2011), and they have just recently begun to be applied to the study of ecological networks (Payrató‐Borràs et al., 2019). This class of null models offers both methodological and conceptual advances, since the theoretical ensemble produced is statistically nonbiased and considers the possibility that the observed network is in fact incomplete. However, while the majority of nestedness metrics have been tested for algorithmically based null models (Almeida‐Neto et al., 2008; Ulrich & Gotelli, 2007), their behavior in maximum‐entropy ensembles is still largely unexplored.

In this work, we focus on the problem of measuring nestedness by presenting a comparative study of the behavior of six nestedness metrics, most of which are commonly included in popular packages and cited in the literature. Our purpose is twofold: First, we aim to test the performance of these metrics under the maximum‐entropy null model recently used in Payrató‐Borràs et al. (2019), and secondly, we intend to critically assess the functioning of each metrics by analyzing its dependencies with network parameters. By doing this, we mean to, first, fill a gap in the literature concerning null models, and second, to provide a practical guide of the advantages and disadvantages of each nestedness metrics. In comparison with previous works that attempted to characterize the performance of nestedness indices (Ulrich et al., 2009), here we analyze at the same time traditionally used metrics as well as some that are still poorly understood, either because they have been proposed just recently or because of their limited popularity. In addition, by examining the behavior of the variants of two of those metrics, we explore the consequences of assuming different normalizations.

To this end, we study the nestedness metrics using the following procedure: For each of the 199 real bipartite networks of our dataset, we build the corresponding maximum‐entropy and maximum‐likelihood ensemble that preserves on average the observed degree sequence (Payrató‐Borràs et al., 2019; Squartini & Garlaschelli, 2011). We then measure nestedness in the ensemble built for each of the real networks according to each of the metrics, and we compare the results with the corresponding nestedness value of the observed network. Secondly, we perform various statistical analyses to determine the relation of each metrics with network properties, such as size, fill, and degree degeneracy. With this information at hand, we finally perform a thorough comparison among the different metrics, evaluating their qualities and flaws. Furthermore, this theoretical analysis is accompanied by a working repository called *nullnest*, which allows reproducing these results and includes, moreover, some key results and examples.

## 2 MATERIALS AND METHODS

### 2.1 The studied metrics

We briefly describe here the principal characteristics of the indices used in this work in order to quantify nestedness. The technical details on how each metrics has been numerically implemented can be found in Appendix S1.

**Figure 3:**
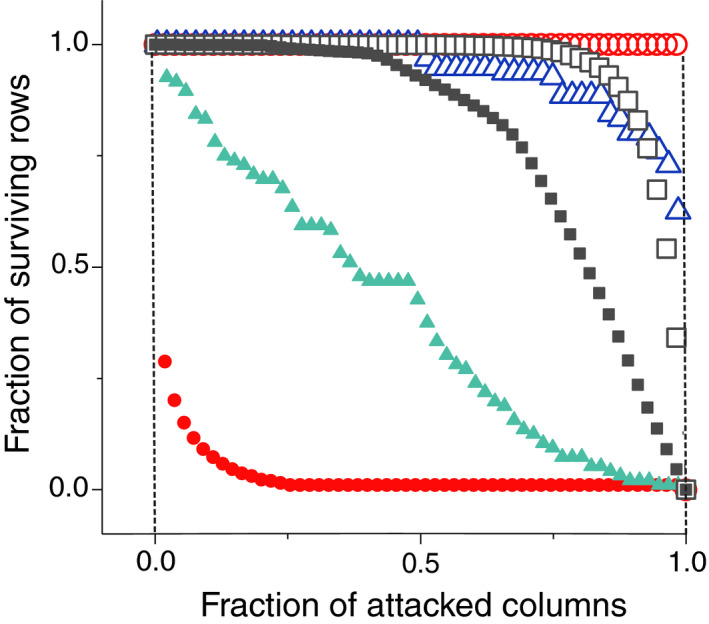
Attack Tolerance Curves for three different networks having the same parameters. Triangles correspond to the real mutualistic ecosystem of Clements and Long (1923), squares to a randomization of this system and circles to an artificial perfectly nested network with the same parameters (size and number of links). Open and full symbols correspond to the IDR and DDR attack strategies, respectively

- *The Atmar and Patterson temperature* (*T*_AP_) (Atmar & Patterson, 1993). This nestedness metrics is based on the idea of quantifying the deviations of a real matrix from a perfectly nested matrix by measuring the distance of the misplaced interactions from the IPN curve (see Figure 2).
  In particular, the mathematical basis of this metrics relies on the mapping of the maximally packed version of a *m* × *n* bipartite adjacency matrix into a continuous rectangle (Medan et al., 2007), leading to the analytic expression of the IPN in terms of two continuous variables $a\in \left[0,\;n\right]$ and $p\in \left[0,\;m\right]$, which constitute the continuous approximation of the discrete labels of the columns and rows of the biadjacency matrix, respectively. This approximation is expected to be correct in the limit of very large systems. Then, the nonzero elements of the biadjacency matrix correspond, in the rectangular surface of size *m* × *n*, to an area proportional to the density of contacts *ϕ* = *E*/(*m* × *n*), where *E* is the total number of edges. This area may be assumed to be colored and so the empty area represents the amount of zero elements of the adjacency matrix. The IPN can be analytically expressed as a function of *m*, *n,* and *ϕ* (Medan et al., 2007).
  Because real systems are not perfectly nested, the *T*_AP_ measures the distance, along the diagonal of the unit square, of the misplaced points (presence or absence of a contact above or below the IPN) (Atmar & Patterson, 1993). Various implementations of this metrics can be found in the literature (Atmar & Patterson, 1995; Guimarães & Guimaraes, 2006; Rodríguez‐Gironés & Santamaría, 2006); however, the most popular nowadays is probably BINMATNEST, developed by Rodríguez‐Gironés and Santamaría (2006). Indeed, in this work, the authors proposed to quantify the *unexpectedness* of a given interaction of the matrix, mapped into the unit square, by the following function (Rodríguez‐Gironés & Santamaría, 2006):
  (1) $$u_{\mathrm{ij}}={\left(\frac{d_{\mathrm{ij}}}{D_{\mathrm{ij}}}\right)}^{2}$$
  where *d_ij_* and *D_ij_* correspond, respectively, to the distance between the unexpected interaction and the IPN in the first case and to the total length of the diagonal in the second (see Figure 2). The final temperature is then calculated as follows:
  (2) $$T_{\text{AP}}=\frac{100}{U_{\max}\cdot n\cdot m}\sum u_{\mathrm{ij}}$$
  where the sum runs over all the unexpected interactions and *U*_max_ is a constant given by Atmar and Patterson (1993). Accordingly, the *T*_AP_ will be large if there are several "1s" and "0s" on the wrong side of the IPN. It will be even larger if those misplaced points are located far from the IPN. Therefore, the lower the measure of the *T*_AP_ of a given system, the more nested it is.
- *The nestedness index based on Manhattan distance (NMD)* (Corso, Araujo, & Almeida, 2008). This metrics follows the same idea as the *T*_AP_ metrics, in the sense that it counts the number of unexpected presences or absences with respect to a perfectly nested matrix of the same characteristics (size and fill) as the studied matrix, when both are brought to their maximally packed form. Again, it introduces a mapping of the matrix into the unit square. However, on this rescaled continuous approximation, it measures the distance to the corner of the matrix where the nested core is expected. Moreover, distances are measured in terms of the Manhattan distance, which means that the distance between a rescaled element *b_i_*_,_ *_j_* of the matrix and the origin is *d_i_* *_j_* = *x_i_* + *y_i_*. Taking this into account, the nestedness index is given by the following:
  (3) $$\tau =\frac{d‐d_{\text{nest}}}{d_{\text{rand}}‐d_{\text{nest}}},$$
  where *d* is the sum over all the elements' distances $d=\sum d_{i,j}$ of the real matrix (maximally packed) and *d*_nest_ represents an analogous sum but over the corresponding perfectly nested matrix with the same size and fill as the empirical one. Their difference is then normalized by the maximum difference in average distances between a null model and the perfectly nested matrix. In this way 0 ≤ *τ *≤ 1, and the smaller *τ* the more nested the system is. Here, we used the implementation of the popular *bipartite* package (Dormann et al., 2009) where the null model used to calculate *d*_rand_ keeps constant size and fill (see Appendix S1 for details).
- *The nestedness metrics based on overlap and decreasing fill (NODF)* (Almeida‐Neto et al., 2008). This index measures the average percentage of shared contacts between pairs of rows which present a decreasing degree ordering (idem for columns). Almeida‐Neto et al. proposed an operational definition for calculating the NODF metrics, that for an *n* × *m* biadjacency matrix *B* has been analytically summed up as follows (Payrató‐Borràs et al., 2019):
  (4) $$\begin{array}{cc} \text{NODF}= & \frac{1}{K}\sum_{i<j}^{n}\left\{\left[1‐\theta \left(d_{j}‐d_{i}\right)\right]\cdot \frac{\sum_{a=1}^{m}b_{\mathrm{ia}}b_{\mathrm{ja}}}{d_{j}}\right\}+\\ & \;\frac{1}{K}\sum_{k<l}^{m}\left\{\left[1‐\theta \left(d_{l}‐d_{k}\right)\right]\cdot \frac{\sum_{p=1}^{n}b_{\mathrm{pk}}b_{\mathrm{pl}}}{d_{l}}\right\} \end{array}$$
  (5) $$\text{where}\;K=\frac{n\left(n‐1\right)+m\left(m‐1\right)}{200}$$
  Here, *d_p_* is the degree of row *p* while *d_a_* is the degree of column *a*. We consider that the matrix *B* is ordered by decreasing degree, and row *i* is placed above row *j* and column *k* at the left of column *l*. The normalization factor *K* accounts for all possible pairs and the fact that NODF is defined to take values between 0 and 100. Finally, the *θ* represents the Heaviside step function. For this metrics, the higher the NODF index, the more nested the system is. Furthermore, NODF has the advantage that it can be calculated not only algorithmically but also by using a closed mathematical expression in terms of the elements of the biadjacency matrix, which allows for analytic studies (Payrató‐Borràs et al., 2019).
  This metrics correctly assigns a very low nestedness value to modular networks (because, in general, elements within the same block have similar degree), but it may give a false negative (a low value) in the case of a nested network with multiple rows (columns) with the same degree (Staniczenko et al., 2013). Unfortunately, this situation is quite common for mutualistic ecosystems which are in general very sparse and often eccentric, with typically much more animal species than plant species, leading to a non‐negligible degree degeneracy. For this reason, a variant of this metrics called *stable‐NODF* has recently been proposed by Mariani et al. (2019). Its analytical expression reads the following:
  (6) $$\begin{array}{c} \text{stable}‐\text{NODF}=\frac{1}{K}\sum_{i<j}^{n}\left(\frac{\sum_{a=1}^{m}b_{\mathrm{ia}}b_{\mathrm{ja}}}{d_{j}}\right)+\frac{1}{K}\sum_{k<l}^{m}\left(\frac{\sum_{p=1}^{n}b_{\mathrm{pk}}b_{\mathrm{pl}}}{d_{l}}\right) \end{array}$$
  (7) $$\text{where}\;K=\frac{n\left(n‐1\right)+m\left(m‐1\right)}{200}$$
  This variant does not incorporate the decreasing fill term and hence does not penalize the degree repetition, therefore solely measuring the number of shared partners among pairs of rows and columns.
- *The Brualdi and Sanderson discrepancy* (Brualdi & Sanderson, 1999). Starting from the real matrix in its maximally packed state, this metrics measures the number of misplaced absences or presences of contacts, called *discrepancies*, that should be "corrected" in order to produce a perfectly nested matrix with equal size and fill. Given that the number of possible discrepant links is directly proportional to the total number of links, the result greatly depends on the network's fill. To hinder the results from such dependency, we normalized the discrepancy by the total number of links, as suggested by Greve and Chown (2006).
  On the other hand, since this metrics is based on the comparison of the real matrix with a perfectly nested one of the same parameters (*n*, *m,* and *ϕ*), it is independent of a particular null model. However, given that there may be some ambiguity on the maximally packed configuration, the result depends on the chosen one. Therefore, the best approach would involve averaging over the different initial maximally packed configurations of the observed matrix, yet this procedure is very demanding numerically and we do not implement it in this work. As it was the case of the temperature *T*, the definition of this metrics implies that the lower the value of the index, the more ordered the system is.
- *The nesting index based on network's robustness (NIR)* (Burgos et al., 2009). The NIR metrics is based on the notion of the robustness of a network, that is, the capacity of the system to remain connected when subject to node removal (Burgos et al., 2007; Memmott, Waser, & Price, 2004). This index uses two extreme node removal procedures, or *attack strategies*, whose outcomes reveal the amount of nestedness of the network. On the one hand, the nodes of one guild are removed in *decreasing degree order* (DDR strategy), and of the other in *increasing degree order* (IDR strategy). The fraction of species of the other guild that still keeps contacts (survive) as the counterparts are removed leads to the *Attack tolerance curve* (ATC). Figure 3 illustrates three different typical behaviors of the ATC for each strategy, when the procedure is applied on a perfectly nested network, on a real network, and on a null model with the same size and fill. The DDR strategy better reveals the differences of structure of the three networks.

It can be easily shown (Burgos et al., 2009) that, for the perfectly nested network, the area under the ATC is *R*_IDR_ = 1 for IDR strategy, while it is *R*_DDR_ = *ϕ*, for the DDR. The index is normalized by the area between these extreme curves, which is maximum for a nested network. Moreover, the area is minimum for a random network, while for the real networks, the area lies between these two extremes. Therefore, the contribution to the nestedness coefficient of rows or columns is defined as follows:

(8) $$\text{NIR}=\frac{R_{\text{IDR}}‐R_{\text{DDR}}}{1‐\phi }$$

which measures, like NODF, the contribution to nestedness of rows and columns separately. NIR looses sensitivity as the density of links increases, which is not a problem for ecosystems that are, in general, very sparse. Finally, this index may in principle slightly depend on the chosen matrix ordering with respect to the degrees of the guild being suppressed. As such order is not unique due to degree degeneracy, in our implementation we have averaged over a set of equivalently ordered matrices.

- *The spectral radius* (Staniczenko et al., 2013). Staniczenko et al. (2013) recently proposed a nestedness metrics based on the spectral properties of double nested graphs. In particular, they relied on a theorem that proves (Bell, Cvetković, Rowlinson, & Simić, 2008; Bhattacharya, Friedland, & Peled, 2008) that among all the connected bipartite graphs of *n* + *m* nodes and with *E* edges represented by its adjacency matrix *A*, the one yielding the largest spectral radius is a perfectly nested graph. Indeed, they showed that more nested graphs *tend* to have a larger spectral radius (though this relation is not monotonous). Interestingly, nevertheless, since this metrics involves diagonalizing a symmetric matrix, it is independent of the ordering of the matrix and does not suffer from the ambiguities of finding the maximally packed form of the bipartite matrix as the previous metrics.

An important drawback of the spectral radius is that it is not normalized. Therefore, we propose to normalize the spectral radius obtained for each system with that of the perfectly nestedness matrix having the same size and fill (see Appendix S1 for details), as was already suggested by Staniczenko et al. That is, if *ρ* represents the spectral radius of a real network and *ρ*_max_ the spectral radius of a perfectly nested graph with the same size and fill, the normalized index *ρ*_norm_ is given by the expression:

(9) $$\rho_{\text{norm}}=100\frac{\rho }{\rho_{\max}}$$

In the next sections, we study both versions of this metrics: the (not normalized) original one along with the normalized modification given by Equation 9.

### 2.2 The maximum‐entropy–maximum‐likelihood realization of the FF null model

We use a null model for bipartite networks that constrains the degree sequence of each guild, so that they are kept only *on average*. That is, at variance with the previously studied FF null model, where the real degree sequences are enforced *strictly* (Ulrich & Gotelli, 2007), in our case the degree sequences of a sampled null network may slightly vary from the real ones, with the restriction that the average degree sequences are preserved. From a theoretical perspective, relaxing such constraints reflects the fact that the observed degree sequences may provide imperfect information, that is, the reported network may contain noisy data like missing or mislead interactions. Importantly, the resulting statistical ensemble is obtained by maximizing its entropy as well as the likelihood of finding the real degree sequences, which leads to the Exponential Random Graph model (Park & Newman, 2004). This randomizing framework was first developed by Squartini and Garlaschelli (2011), then extended to bipartite networks by Saracco, Di Clemente, Gabrielli, and Squartini (2015). We also applied it to the study of the emergence of nestedness in ecological networks in Payrató‐Borràs et al. (2019).

In order to maximize the entropy under the condition of keeping the average degree sequence fixed and equal to the observed one, it is necessary to apply the Lagrange Multipliers' technique. The determination of the value of such multipliers, obtained by maximizing the likelihood that the empirical degree sequence appears in the random ensemble (Garlaschelli & Loffredo, 2008), provides an *analytic expression* for the probability of interaction among species or agents from different guilds. Remarkably, this probability only depends on the Lagrange Multipliers. These variables, in turn, can be determined by computationally solving the optimization problem of the likelihood. We have used two different numerical methods to find the global optimal Lagrange Multipliers for each empirical network: (a) a global searching algorithm based on simulated annealing, and (b) a local optimization method repeated over a variety of initial conditions. The technical details of the implementation of these techniques can be found in Payrató‐Borràs et al. (2019). We performed both analyses for each of our real networks and verified that the results agree, which is a strong indication that the global maximum has been found.

This probabilistic bipartite matrix can then be used to sample the random ensemble of networks. In particular, for each real network in our dataset, we sampled 10^4^ null networks with the obtained probability interaction matrix. Across the same sample, each of these null matrices may vary in its size (number of connected nodes), density of links, degree sequence, redundancy of degrees, or bipartite matrix eccentricity. Nevertheless, the degree sequences are maintained, on average, equal to the empirical ones.

The present null model overcomes many of the statistical bias exhibited by the FF null model. A first conceptual difference between the present and previous null models is that the resulting ensemble of networks is treated from a statistical physics perspective, that is, by using the probability of appearance of each network in the ensemble. Moreover, loosing the constraint of preserving exactly the degree sequences yields to an expansion of the statistical ensemble. On the contrary, with the FF null model the number of null networks compatible with the constraints becomes scarce whenever the real network we aim to randomize is significantly small, dense, or nested. On the other hand, the double maximization of the entropy and the likelihood produces a maximally disordered statistical ensemble while constraining certain information extracted from real systems, in our case the empirical degree sequences. Garlaschelli and Loffredo (2008) showed that such construction is statistically nonbiased. Finally, the fact that our null model provides an analytic expression for the probability of interaction between species results in the computational generation of null networks being fast, efficient, and demanding few numerical resources. Instead, the FF null model relies on an algorithmic procedure that can easily become frustrated, thus slowing down the production of null networks and being computationally demanding. All these advantages, together with the conceptual basis argued above and in (Payrató‐Borràs et al., 2019), lead us to choose this null model to assess the performance of a variety of nestedness metrics.

### 2.3 Dataset

Our study has been carried out using a large dataset composed by a total of 199 bipartite networks. In particular, it includes 191 empirical ecological networks extracted from the *Web of Life* (Bascompte Lab) as well as eight economic networks which represent the trading interactions between the buyers and the sellers of two different fish markets (Hernández et al., 2018). A more detailed description of these systems may be found in Appendix S1. All the networks in our dataset were treated as binary (nonweighted links). We have only kept in our study networks with a minimum size of 20 nodes.

### 2.4 The *nullnest* repository

Additionally to the comparative study here presented, we provide an open github repository named *nullnest* that aims at being a practical tool, for both ecologists and network scientist, to assess the nestedness of real and null networks. The repository is thoroughly documented, with examples and ready‐to‐use programs, and allows performing the analysis discussed in the present paper as well as some of the main calculations derived in Payrató‐Borràs et al. (2019). It can be freely accessed and downloaded at https://github.com/cclaualc/nullnest.

## 3 RESULTS

### 3.1 Significance of nestedness of empirical networks

To start with, we have measured the nestedness of the 199 empirical networks in our dataset. To compare the average nestedness over the ensemble with that corresponding to empirical networks, we have used the six metrics described above plus two variations (the stable‐NODF and the normalized spectral radius).

As it has been shown analytically and numerically (using NODF and the spectral radius) in Payrató‐Borràs et al. (2019), the nested structure of mutualistic networks is a consequence of the double heterogeneity in the degree sequence which results from entropic effects. In order to investigate if the other popular indices are able to reveal this dependence of the nestedness on the degree distributions, we built a null model for each real network, as explained in Section 2.2, and we compared the nestedness of each real network with its corresponding average over the ensemble. For each of the studied metrics, the average value of nestedness over the randomized ensemble has been obtained by numerical sampling as described in Appendix S1.

For the sake of clarity and to homogenize the reading of the diverse figures, we have transformed the definition of the temperature, the NMD, and the discrepancy indices so that the larger the index, the more nested the system is. We have also rescaled these indices so that they vary between 0 and 100. These modifications read as follows:

(10) $$T=100‐T_{\text{AP}}$$

(11) $$\text{NMD}=100\left(1‐\tau \right)$$

(12) $$\Delta^{\prime }=100\left(1‐\frac{\Delta }{E}\right)$$

where *τ* corresponds to the original metrics based on Manhattan distance and Δ to the original discrepancy index (see section 2.1), while *E* is the total number of edges in the network.

**Figure 4:**
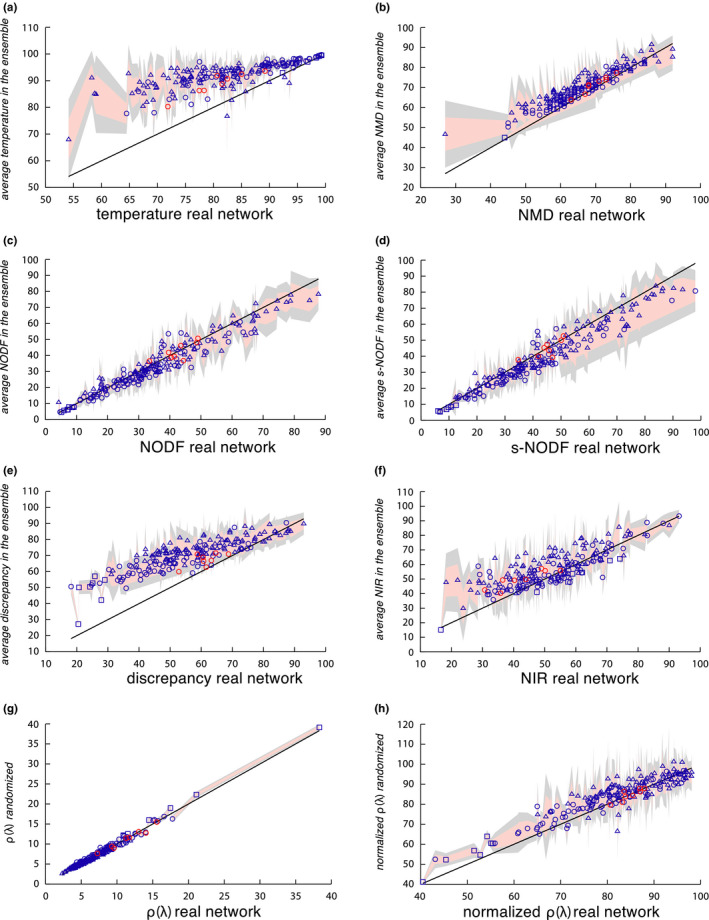
Significance of the nestedness of real networks. The figure shows the empirical measure of nestedness against the average value of nestedness in the generated statistical ensemble for the 199 empirical networks in our dataset. The different panels correspond to different metrics: (a) temperature, (b) NMD, (c) NODF, (d) stable‐NODF, (e) discrepancy, (f) NIR, (g) spectral radius, and (h) normalized spectral radius. The shadowed areas represent one (salmon color) and two (light gray) standard deviations of the mean. The black line depicts the identity curve. Triangle symbols stand for small networks (less than 50 nodes), circles for medium size networks (more than 50 nodes and less than 410), and squares for large networks (more than 410 nodes). Ecological networks are colored in blue, economic networks in red

Figure 4 shows the nestedness measured over the ensemble versus the nestedness of the corresponding real network. Consistently with the results obtained in Payrató‐Borràs et al. (2019) using NODF and the spectral radius, NIR and NMD also show that the nestedness values of the empirical networks are statistically equivalent to the average of the corresponding randomized ensemble. This leads to the conclusion that the observed nestedness measured by these indices is not significant. On the contrary, the discrepancy and temperature indices show a clear bias, with an important fraction of the real networks being *less* nested than the random average.

### 3.2 Influence of network properties on the behavior of the different metrics

**Figure 5:**
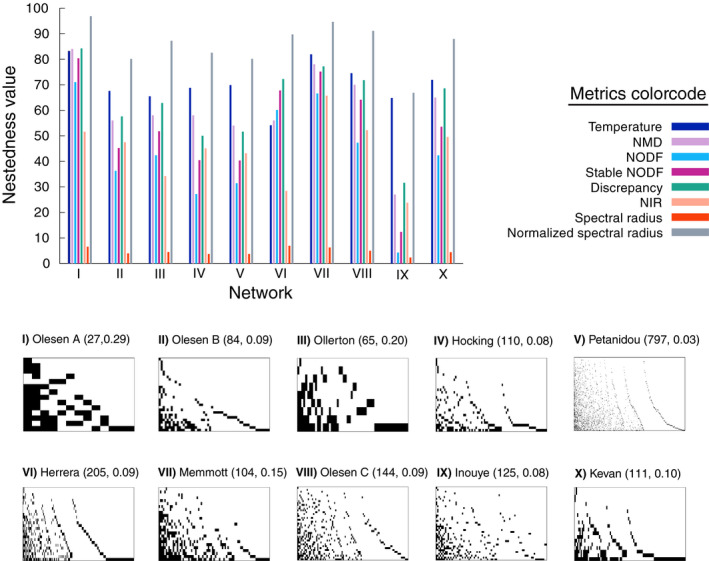
Comparison among nestedness indices. The histogram on the top of the figure shows how eight different metrics measure the nestedness of several different networks. Each network, indexed I to X, is represented in the bottom of the figure by its biadjacency matrix ordered by decreasing degree, with the interactions among species represented by black pixels. All networks represent plant–pollinator mutualistic communities extracted from the Web of life dataset (Bascompte Lab). Each network is labeled with the name of the first author of the corresponding reference, followed within brackets by, first, its total number of species (number of plants plus number of animals), and second, its density of links

The results presented in Figure 4 reveal that the metrics studied behave in different ways under the same null model, showing distinct levels of fluctuations and sometimes a systematic bias, as it is the case for the discrepancy and temperature indices. This finding suggests that the different algorithms implemented by each metrics may eventually translate into nonequivalent nestedness measures. We explore further this situation in Figure 5, where we compare the values of nestedness obtained for a group of mutualistic networks when measured using each of the metrics. As it can be observed, for the same dataset not only the value of nestedness itself but also the ranking of the networks according to their degree of nestedness is strongly metrics dependent.

Ideally, as it has been recalled by several authors (Almeida‐Neto et al., 2008; Staniczenko et al., 2013; Ulrich et al., 2009), a well‐behaved nestedness metrics ought to be independent of the particular network parameters and, furthermore, rank the degree of nestedness of a given set of networks *universally*. The results discussed above put in evidence that the second condition is not always true. Regarding the first requirement, we next explore more carefully how the nestedness values given by each metrics depend on the network parameters. In particular, since the networks of the dataset cover a wide range of parameter values (see Figure 5 for an example), we analyze the effects of three characteristic network properties: size, density of links, and eccentricity. These quantities are defined as follows:

(13) size≡s=n+m

(14) $$\text{density}\;\text{of}\;\text{links}\equiv \phi =\frac{E}{n+m}$$

(15) $$\text{eccentricity}\equiv \epsilon =\left|\frac{n‐m}{n+m}\right|$$

where *n* and *m* are, respectively, the number of rows and columns of the biadjacency matrix, while *E* is the total number of links. The eccentricity quantifies the difference between the number of nodes of the two guilds, or in other words, the deviation from a square‐shaped biadjacency matrix. Indeed, ϵ = 0 for a square matrix and ϵ → 1 when one of the guilds is much larger than the other. Interestingly, most of the large ecological networks observed show more columns (animal species) than rows (plant species), with a frequent ratio of 1–3. This observation, though, cannot be generalized to all mutualistic networks, specially to small networks (which can be much more eccentric) or to nonecological systems.

Additionally, we study the dependence of nestedness on a fourth parameter, the degree degeneracy. In particular, a perfect nested matrix with an arbitrary *ϕ* might have several species of each guild with the same degree. We measure this quantity as follows:

(16) $$\text{degeneracy}\;\text{in}\;\text{degrees}\equiv g=\frac{\text{number}\;\text{of}\;\text{species}\;\text{with}\;\text{the}\;\text{same}\;\text{degree}}{n+m}$$

The study of this parameter remains a special case, since the known connection between the nested patterns and the degree sequences entails that a certain dependency with the degree degeneracy is in fact expected (Jonhson et al., 2013; Payrató‐Borràs et al., 2019). All in all, we analyze its influence given that each metrics deals with degree degeneracy in a different way.

**Figure 6:**
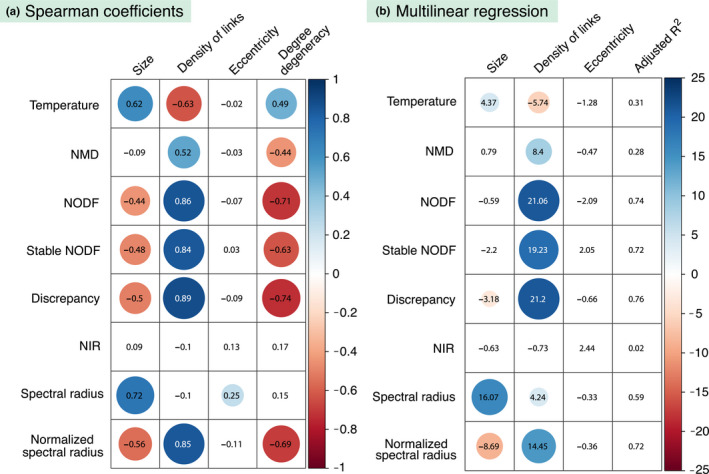
Dependency of nestedness metrics on network parameters. The left panel, (a), shows the Spearman correlation factor between the network parameters (columns) and the eight nestedness metrics under study (rows). The numbers represent the value of the Spearman rank coefficient for each corresponding pair of nestedness value and network parameter. Only those coefficients that are statistically significant (*p*‐value < .01) are highlighted by a colored circle, being the size and the color of the circle proportional to the coefficient. The right panel, (b), summarizes the results of the multilinear fit detailed in Equation 17. Each row corresponds to a different nestedness metrics. The first column from the right shows the adjusted coefficient of multiple determination (adjusted *R* ^2^). The other three columns show the *t*‐ratio of the regression coefficient corresponding to each explanatory variable (as labeled by the column name). Only those coefficients that are statistically significant (*p*‐value < .01) are highlighted by a colored circle, being the size and the color of the circle proportional to its *t*‐ratio

In order to quantify the dependencies discussed above, we have performed a twofold analysis. First, we have calculated Spearman's rank correlation between the nestedness index given by each metrics and the different network parameters. This coefficient allows to assess the relation between both variables without assuming a linear behavior. Figure 6a summarizes the result of the analysis, showing the Spearman coefficient along with its statistical significance for all pairs of nestedness values and network parameters (see Appendix S1 for the details on the numerical calculation). Secondly, we have performed a multilinear regression. In particular, we have taken the nestedness values obtained by each metrics as the dependent variable while the network parameters behave as the explanatory variables. Importantly, in this second analysis we do not consider the effect of the degree degeneracy, since we are mainly interested on the dependence on parameters that should *not*, in principle, determine nestedness. The linear function we have fitted has the following standard form:

(17) $$\nu_{j}=\beta_{0,j}+\beta_{1,j}s+\beta_{2,j}\phi +\beta_{3,j}\epsilon +\varepsilon$$

where *ν_j_*, *j* = 1,…,8 represents the nestedness metrics indexed by *j*, *β*_0,_ *_j_* is the intercept and *β_i_*_,_ *_j_*, *i* = 1,…,3 are the partial regression coefficients. The *ε* represents an error term. This sort of regression informs on the effect of a single network parameter when the rest of parameters are kept fixed. Such consideration is specially important given that, in natural systems, networks' properties are often correlated (for instance, larger networks tend to be less dense), and therefore, bivariate regressions may misleadingly quantify the influence of a certain property due to the uncontrolled coupled influence of another one. On the other hand, our model assumes a linear relation among the variables which might not always be accurate. Figure 6b shows the results of the regression for each nestedness metrics, in particular, the significance of the partial coefficients corresponding to the different network parameters as well as the value of the adjusted coefficient of multiple determination (see Appendix S1 for more details).

**Figure 7:**
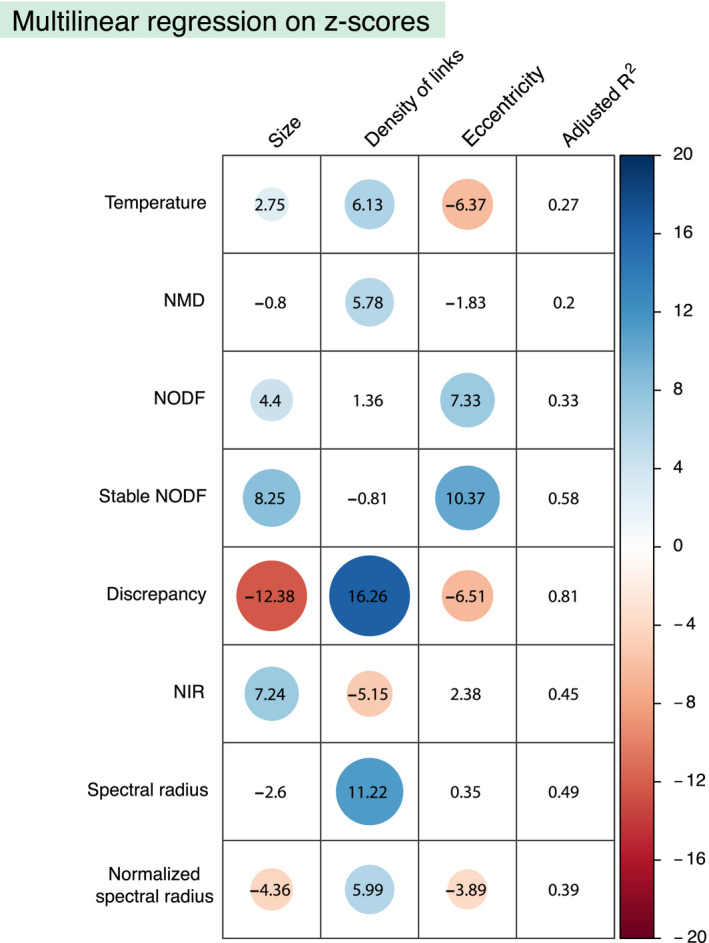
Dependency of *z*‐scores on network parameters. The figure summarizes the results of a multilinear regression between the *z*‐scores values corresponding to each nestedness metrics and network properties. Each row corresponds to the *z*‐scores obtained by applying to each metrics the null model discussed in section 2.2. The first column from the right shows the adjusted coefficient of multiple determination (adjusted *R* ^2^). The other three columns show the *t*‐ratio of the regression coefficient corresponding to each explanatory variable (as labeled by the column name). Only those coefficients that are statistically significant (*p*‐value < .01) are highlighted by a colored circle, being the size and the color of the circle proportional to the *t*‐ratio

Once we have quantified the dependencies of the various nestedness metrics on different network parameters, we next explore whether we can explain the deviations with respect to the null model observed in Figure 4. In particular, we perform a multilinear fit of the type detailed in Equation 17, where we replace the nestedness values by the *z*‐scores obtained for each metrics when applying the null model discussed in sections 2.2 and 3.1. Such *z*‐scores are calculated as follows:

(18) $$z{\;‐\;\text{score}}_{j}=\frac{\nu_{j}‐\left\langle \nu_{j}\right\rangle }{\sigma_{j}}$$

where *ν_j_* represents, as before, the real values obtained with a nestedness metrics indexed by *j*; 〈*ν_j_*〉 represents the average nestedness value calculated with metrics *j* over the null ensemble ; and *σ_j_* represents the standard deviation of the distribution of nestedness in the ensemble for the same metrics. By fitting a linear function analogous to Equation 17, we obtained, thus, the partial coefficients which account for the contribution of each network parameter to the corresponding *z*‐scores. A summary of these results can be found in Figure 7.

## 4 DISCUSSION

In the previous section, we have quantified the influence of several network properties on various nestedness metrics, taking into account how each metrics measures the nestedness of empirical networks as well as how they compare to the null model of section 2.2. With this information at hand, we now proceed to critically evaluate the performance of each metrics by discussing and framing the observed dependencies in a general context.

### 4.1 Temperature

Despite its popularity, this metrics was already known to have several flaws (Almeida‐Neto et al., 2008) and various authors have outlined the presence of ambiguous steps in its calculation (Mariani et al., 2019; Rodríguez‐Gironés & Santamaría, 2006). Indeed, Almeida‐Neto et al. (2008) called upon its dependency on the density of contacts, *ϕ*, and on the size of the matrix. We confirmed these dependencies since our statistical analysis shows that real values correlate positively with size and negatively with the density of links (see Figure 6). Interestingly enough, the temperature is as well the only metrics to show a significant positive correlation with the degree degeneracy, while the rest of metrics penalize the repetition of degrees.

Moreover, when tested against the null model, the temperature exhibits a clear bias (see Figure 4). In fact, the average nestedness in the ensemble is systematically larger than the real observations. The multilinear regression performed using the *z*‐scores shows that they correlate significantly with the size, the density of links, and the matrix eccentricity. As shown in Figure 4, the bias of this metrics shows a negative *z*‐score value; therefore, its modulus (which gives the relative distance to the identity curve) increases when the size of the network is smaller, less dense, and more eccentric. Given that mutualistic ecological networks usually present low density and a pronounced eccentricity, these conclusions point out that the temperature metrics should be applied, if at all used, with care in the ecological context.

### 4.2 Nestedness index based on Manhattan distance

Our analysis of the nestedness metrics based on the Manhattan distance shows that it correlates positively with size and negatively with the degree degeneracy. Interestingly, the deviations with respect to the null model are sensibly smaller than in the temperature metrics, though a slight but systematic positive deviation still appears leading again to a negative *z*‐score. The multilinear regression indicates that the *z*‐scores are mainly explained by the density of links, which have a positive influence meaning that denser networks fall closer to their null expectation.

Overall, the NMD metrics exhibits notably less dependencies than its close metrics temperature, which with the NMD shares a common spirit given that both metrics measure somehow the distance of unexpected interactions. This dissimilarity is probably due to the different normalization of the NMD. On the other hand, such normalization is dependent on the null model used (Corso et al., 2008), and hence, the metrics is inevitably subject to the same limitations (see Appendix S1 for more details on the implementation of NMD, in our case using the FF null model).

### 4.3 NODF and stable‐NODF

In the work in which the NODF metrics was firstly proposed, Almeida‐Neto et al. (2008) found a positive dependency with the matrix fill. In our analysis, we recover this result and observe as well a negative correlation with the network size (see Figure 6a) which is nonetheless a veiled consequence of the variation in the density of links, as can be understood after performing the multilinear regression (see Figure 6b). Furthermore, this nestedness index exhibits a good agreement with the null prediction, as was already found in Payrató‐Borràs et al. (2019). The differences with the null model, quantified by the *z*‐score, are explained mainly by the size and the eccentricity. As expected from a statistical point of view, the small and eccentric networks show the largest difference with the null expectation.

Although the NODF metrics is nowadays extensively used, some authors have raised a few concerns about its adequacy. In particular, Staniczenko et al. (2013) criticized the decreasing fill factor in its definition, which penalizes degree degeneracy. Indeed, we do observe a strong negative correlation with degree degeneracy for NODF in Figure 6a. As a solution, Mariani et al. (2019) proposed an alternative version of the metrics called stable‐NODF, which does not incorporate this decreasing fill. Our analysis determines that dependencies of both versions of the metrics are very similar: On the one hand, the stable‐NODF does moderate the correlations exhibited by NODF both on degree degeneracy and density of links, but on the other hand, the correlations of the *z*‐scores with the size and eccentricity are strengthened.

### 4.4 Discrepancy

The discrepancy index shows a significant dependency on the size and the density of links, being the latter parameter the dominant one as it can be seen from the multilinear regression (see Figure 6). These dependencies had been noted already (Almeida‐Neto et al., 2008). Interestingly, these findings are very similar to the correlations observed for NODF and stable‐NODF, despite the fact that the metrics are based on distinct strategies for measuring nestedness.

On the other hand, the test against the null model reveals that for an important fraction of networks, the real value of nestedness is smaller than the average in the ensemble, resulting in a systematic deviation with negative *z*‐scores. This shift is very well explained by the regression of the *z*‐scores summarized in Figure 7, where it can be observed that the three network parameters studied correlate significantly with the *z*‐scores. Indeed, the larger, less dense, and more eccentric the network, the more distance there is between the null expectation of nestedness and the empirical value.

### 4.5 Nesting index based on network's robustness

The nestedness index based on network robustness exhibits no dependencies on the network parameters. Indeed, our statistical analysis reveals no significant correlation with any of the studied properties (see Figure 6). This suggests that, despite not being particularly popular, the NIR metrics is a reliable option for measuring nestedness. At the same time, the analysis done using the null model indicates that the nestedness value of smaller and denser networks tends to fall further apart from their null expectation. Indeed, this is a consequence of its definition, which relies on the difference between the areas of the ATCs obtained by the DDR and IDR node removal strategies. As the curvature of the former reproduces the shape of the IPN, it becomes less convex as the density increases, leading to a loss of sensitivity. Therefore, this metrics is well adapted for ecological networks that usually show low densities, but less suited for other bipartite networks, like the aggregated market networks.

### 4.6 Spectral radius

Among the metrics described, the spectral radius shows a significant dependency on both the size and the density of links, specially the former one. Indeed, larger, and denser networks tend to have a larger spectral radius. This is a consequence of the lack of normalization, as mentioned in section 2.1. However, the spectral radius shows a remarkable agreement between the average over the ensemble and the value of the corresponding real network, along with a very low dispersion. Nonetheless, the *z*‐scores correlate significantly well with the density of links, being the most denser networks the ones that exhibit a larger discrepancy with the null model.

In order to hinder the strong dependency on the network size, we evaluate as well as normalized version of the spectral radius, as explained in section 2. Taking into account this normalization, it is now possible to compare the degree of nestedness of networks of different sizes, and to study how different network parameters affect the nestedness index. We find that this normalized version of the spectral radius correlates positively with the density of links, and negatively with the size and the eccentricity of the matrix. Notably, these dependencies are analogous to the ones shown by the NODF, stable‐NODF, and discrepancy indices. At the same time, the analysis against the null model reveals a slight deviation toward a larger value of the average in the random ensemble with respect to the empirical value. This deviation is stronger for larger, less dense, and more eccentric networks.

Besides the mentioned dependencies, when using the spectral radius, it is essential to consider its underlying basis for measuring nestedness. As we pointed out in the introduction, the relation between the spectral radius and the degree of nestedness is not strictly monotonic, but only holds on statistical terms. This hampers its usefulness to rank networks according to their nestedness.

## 5 CONCLUSIONS

Although it has recently been shown that nestedness is not an emergent irreducible pattern of the network, it still remains an interesting quantity to measure, since it constitutes a global property that informs on the heterogeneity of the degree distributions of the guilds. This is particularly relevant for ecological networks because of their typical, rather small sizes preclude a correct fit to a fat tail distribution, like a power law, on the available data. Because of the interest among network scientists for this pattern, specially in ecology, different definitions of nestedness coexist in the literature. These metrics usually quantify some property of the network following a precise protocol, leading to *operational definitions*. Moreover, several of these metrics are integrated into packages widely used to assess the nestedness values of different networks. Nonetheless, the lack of a unique definition generates confusion when it comes to the comparison between the nestedness values of different networks.

In this work, we have performed a systematic comparative study of the performances of six different metrics and the variants of two of them, addressing their dependency on various network parameters. Based on a large database of real systems, our results clearly put in evidence that the different metrics show diverse dependencies on size, density of contacts, eccentricity, and degree degeneracy. Therefore, if the same group of networks is ranked according to their nestedness, the outcome will depend on the metrics used. Understanding these dependencies for each metrics has helped us to explain, as well, the systematic shifts between the real values of nestedness and the average over a null model based on a maximum‐entropy, maximum‐likelihood ensemble.

The nestedness metrics studied here may be roughly classified in three groups according to the properties of the networks that are used to define them: (i) nestedness metrics based on the number of misplaced elements in the bipartite adjacency matrix with respect to a perfectly nested matrix, like discrepancy; (ii) nestedness metrics based on global properties of the network like, NODF, NIR, and *ρ;* and (iii) nestedness metrics like *T* and NMD that operate similarly to (i) but weighting the distance of misplaced interactions to their ideal location in the perfect nested matrix. Our results point out that the NIR index is, by far, the most independent metrics with respect to the considered network parameters, although it suffers from a lack of sensitivity when the density of contacts is high. Moreover, the NODF, the stable‐NODF, the discrepancy and the normalized spectral radius all show very similar dependencies, that is, a positive correlation with the density of links and, for the latter two, a negative correlation with the size. While a dependency with the size is undesired and ought not to appear when using a proper normalization, some authors have claimed that a positive correlation between nestedness and fill is in fact expected (Almeida‐Neto et al., 2008).

Our work aims at providing a useful guide addressed at practitioners that compiles the different characteristics, advantages, and drawbacks of the most popular nestedness metrics, including recently proposed indices that had not been thoroughly analyzed up to date. We also extended the use of maximum‐entropy‐based null models (Payrató‐Borràs et al., 2019; Saracco et al., 2015; Squartini & Garlaschelli, 2011) to these metrics. Finally, this work is accompanied by a package that allows to calculate all the nestedness indicators studied, generate the null ensemble for any network, as well as a database with the already calculated probabilities allowing to generate the null models for the 199 networks studied here. Given that the interest in studying nested patterns has not ceased to increase within and beyond ecology permeating as well other areas of complex systems as varied as economy, sociology, or anthropology, this updated guide may be useful to any network scientist wondering how to measure nestedness.

## CONFLICT OF INTEREST

The authors declare no competing interests.

## AUTHOR CONTRIBUTIONS

**Clàudia Payrató Borràs:** Conceptualization (equal); Formal analysis (lead); Software (lead); Visualization (lead); Writing‐original draft (equal). **Laura Hernández:** Conceptualization (equal); Funding acquisition (lead); Supervision (lead); Writing‐original draft (equal). **Yamir Moreno:** Conceptualization (equal); Funding acquisition (lead); Supervision (lead); Writing‐original draft (supporting).

### OPEN RESEARCH BADGES

This article has earned an Open Materials Badge for making publicly available the components of the research methodology needed to reproduce the reported procedure and analysis. All materials are available at [Github repository: https://github.com/cclaualc/nullnest; Dryad permanent link: https://doi.org/10.5061/dryad.bk3j9kd8b (stil unactive); and temporary link for peer‐review: https://datadryad.org/stash/share/oTltKtd6i0kDMXMdI4q4WFv_Bn-fwtsykt4QlTHvfKs].

## Supporting information

App S1

## Data Availability

The codes used for the analysis presented throughout this paper, as well as the main results of the null model concerning the real networks studied, are public as a *github* repository under the name *nullnest*, available at https://github.com/cclaualc/nullnest and also in Dryad at https://doi.org/10.5061/dryad.bk3j9kd8b. The dataset of real networks analyzed is already public in the Web of Life site (Bascompte Lab) and a figshare repository (Hernandez, Vignes, & Saba, 2018).
