# Supplementary material for: Measuring nestedness: A comparative study of the performance of different metrics
Source: Ecol Evol. 2020 Oct 22;10(21):11906–21. doi: 10.1002/ece3.6663 (PMC7663079; doi:10.1002/ece3.6663)
Supplement: Supplementary file 1 — App S1 [file ECE3-10-11906-s001.pdf]

SI1: Dataset

Here we provide detailed information about the 199 bipartite networks we analyze. The ecological networks were extracted from the Web of Life repository [1], which contains the following data:

- 118 Plant-pollinator networks. Here, the links among guilds represent mutualistic relationships, characterized by benefiting both interacting agents. In this case, animals, including mainly insects, pollinate flowering plants. This activity provides the pollinators with nutrients while pollinated plants enhance its reproductive success. The two set of nodes of the bipartite network represent the species of the plant and pollinator guilds.

- 23 Seed-disperser networks. Here, the links also represent mutualistic interactions, consisting now of birds feeding on the fruits of certain plants and then contributing to their reproduction and dispersal by disseminating their seeds. Hence, one guild is formed by the plant species and the other by the bird species.

- 43 Host-parasite networks. Here, the links depict a parasitic relationship, where one of the species obtains benefits in detriment of the other. Explicitly, these networks are formed by different flea species which feed on diverse mammal species. Although this is not a mutualistic interaction, the system may still be represented by a bipartite network where the two guilds correspond to flea and mammals species.

- 4 Plant-herbivore networks. Here, the links represent a consumer-resource interaction between insect species (one guild) and plant species (the other guild). In detail, the networks depict different communities where macrolepidopteran species feed on several Prunus species.

- 3 Plant-ant networks. These networks include two examples of diverse types of communities: a network depicting ants which feed on plant nectar, this being a consumer-resource interaction, and two networks representing communities where ant species live in a mutualistic association with certain plant species known as Myrmecophytes.

The economic networks, which are publicly available in [2], consist of:

- 8 economic networks representing buyers-sellers interactions in the Boulogne-sur-Mer Fish Market in France [3]. These are mutualistic networks taken from a very different context. Each network describes the transactions observed in different days in the bilateral or in the auction Fish Market. These daily networks are typically much denser than ecological ones.

SI2: Computation of the nestedness index for each of the studied metrics

Temperature

We calculated the temperature metrics using the *R* software [4] and, specifically, the *bipartite* package [5, 6] version 1.13.0. In particular, we used the *nested* function and we set as method the *binmatnest2* option. This calculates the temperature metrics by using an implementation by Jari Oksanen [7] of the *binmatnest* program by Miguel Rodriguez-Girones [8]. We have redefined the resulting temperature in order to uniform the interpretation of the values yielded by all the metrics such that the higher the value of the corresponding index, the higher the nestedness.

NMD

We calculated the nestedness metrics based in the Manhattan distance (NMD) using the *R* software [4]. We used the *nestedness.corso* function (currently deprecated) from the *bipartite* package [5, 6] version 0.90. For each measure (both for the real networks and the sampled networks), we set to 500 the number of null networks that eventually permits evaluating the significance. Again, we redefined the index to simplify the interpretation of results.

NODF

We wrote a program in FORTRAN90 that computes the NODF and stable-NODF metrics for the real networks, as well as for the corresponding set of null networks.

Importantly, when performing the calculations over the sample of null networks, we kept the same normalization for all sampled networks. That is, we divided the number of overlapping connections, calculated for each null network, by the original number of rows and columns, independently of whether some of the nodes came to have zero degree in the null network.

Discrepancy

We computed the discrepancy metrics using the *R* software [4] and the *bipartite* package [5, 6] version 1.13.0. In particular, we performed the calculation using the method *discrepancy* from the *nested* function.

The final nestedness value measured is directly proportional to the density of links and size of the network. With the aim of removing such dependencies, we divided the resulting value of the metrics by the total numbers of links. This results in a relative discrepancy. Once again, we finally rescaled the index in order to obtain the same monotonic variation for all the indices.

NIR

We implemented a program in FORTRAN90 that calculates the NIR value of the real network and of the corresponding set of null networks. In each case, the resulting value of nestedness is multiplied by 100 in order to preserve the same scale for all the metrics.

In order to account for the possible effects of the degeneracy in the ordering, that is, the fact that multiple configurations are possible when we order rows and columns by their degree, we computed the resulting NIR as the average over a large number of equivalently ordered configurations. These configurations were produced by randomly swapping the matrix position of nodes with the same degree. In more detail, to generate a new ordering we run over all the nodes with degenerate degree and, for each node, we accept a position swap with probability $\frac{1}{2}$.

For each real network, we calculated the degeneracy, *ideg*, the number of repeated degrees. Then, we produced a total of $10 \cdot ideg$ configurations with the same degree order but diverse row and column positions. This procedure was carried out both for the real network and for each null network in the sampling with the exception of the Robertson's network [9] for which, due to its very large size (1500 species), only 10 degenerate configurations have been computed.

Spectral radius

We computed the largest eigenvalue using the *R* software [4], in particular the *eigs_sym* function from the *rARPACK* package [10].

In order to calculate the normalized version of this metrics, we need an estimation of the largest spectral radius of a perfectly nested network of the same size and fill. To estimate each of these values, for each real network in our dataset we produced 100 new networks, characterized by being perfectly nested. These networks were generated using the SNM algorithm [11], which preserves the number of connected nodes (network size) and links, but modifies the pattern of connections and the degree sequences. This algorithm is divided into two procedures. First, the real network is randomized preserving only the fill and the size (that this, ensuring that every node has at least one connection). Second, the SNM algorithm is performed, which consist of iterating the following rules:

(i) We attempt to modify a link by proposing a new partner, randomly selected but different to the original node. The rewiring is susceptible of being accepted only if the new partner has a larger degree than the previous one. This step performs a static version of preferential-attachment.

(ii) If the proposed reconnection leaves one of the nodes with zero degree, the move is discarded. This ensures that the number of connected nodes does not change, thus preserving the network size.

By iterating over these steps *i* and *ii*, one generates a new matrix which is more nested as well as more heterogeneous in its degree sequences than the original one (see [12]). The iteration stops when no more moves are allowed. However, given condition *ii*, this process is not unique and might end up in multiple perfectly nested configurations. To handle this, we generated several optimal configurations per each real network. Specifically, we generated 100 new networks for each empirical network, and exceptionally, for computational reasons, 50 networks for the very large Robertson network. This means that the normalized spectral radius is actually calculated as:

$$\rho_{\text{norm}} = 100 \, \frac{\rho}{\sum_i^{N_{\text{perf}}} \frac{\rho_{\text{perfect},i}}{N_{\text{perf}}}}, \tag{1}$$

where ρ is the spectral radius of the real network and $\rho_{\text{perfect},i}$ represents an optimal configuration with the same size and fill of the real network, produced by the SNM algorithm. N_{perf} corresponds to the number of perfectly nested generated, that in general we set to 100.

When sampling the ensemble, we generated 10 perfectly nested networks per each null network ($N_{\text{perf}} = 10$), and in order to keep the calculations computationally feasible we reduced the sampling size to 500 null networks ($N_{\text{samp}} = 500$). Accordingly, the average normalized spectral radius is calculated as:

$$\langle \rho_{\text{norm}} \rangle = 100 \sum_j^{N_{\text{samp}}} \frac{\rho_{\text{null},j}}{N_{\text{samp}} \sum_i^{N_{\text{perf}}} \frac{\rho_{\text{perfect},i,j}}{N_{\text{perf}}}}, \tag{2}$$

where $\rho_{\text{null},j}$ represents a null network sampled from the statistical ensemble and $\rho_{\text{perfect},i,j}$ represents a perfect configuration produced with the SNM algorithm, having the same size and fill as the corresponding null network.

SI3: Statistical calculations

The statistical correlations were numerically calculated using Python. The Spearman rank correlation coefficient r_s and its p-value were calculated using the Scipy package [13], in particular the *scipy.stats.spearmanr* function.

We performed the linear fits using the *Statsmodels* package [14], which carries out a multilinear least-square regression and provides multiple information, including the adjusted R^2, the partial regression coefficients, their standard deviation and their associated p-value. The t-ratio$_{i,j}$ corresponding to each partial regression coefficient, $\beta_{i,j}$, is calculated as follows:

$$t - \text{ratio}_{i,j} = \frac{\beta_{i,j}}{\sigma_{i,j}} \tag{3}$$

where $\sigma_{i,j}$ is the standard deviation associated to that coefficient. This index provides, hence, information on how significantly different from zero is a certain regression coefficient.

References

[1] Bascompte Lab, *Web of Life, ecological networks database.* Available at http://www.web-of-life.es/.

[2] L. Hernandez, A. Vignes, and S. Saba, *Figshare dataset*, 2018. Available at: https://figshare.com/articles/data_used_for_article_zip/6080396/1.

[3] L. Hernández, A. Vignes, and S. Saba, "Trust or robustness? an ecological approach to the study of auction and bilateral markets," *PloS one*, vol. 13, no. 5, p. e0196206, 2018.

[4] R Core Team, *R: A Language and Environment for Statistical Computing.* R Foundation for Statistical Computing, Vienna, Austria, 2013.

[5] C. F. Dormann, J. Fründ, N. Blüthgen, and B. Gruber, "Indices, graphs and null models: analyzing bipartite ecological networks," *The Open Ecology Journal*, vol. 2, no. 1, 2009.

[6] C. F. Dormann, B. Gruber, and J. Fruend, "Introducing the bipartite package: Analysing ecological networks.," *R News*, vol. 8, no. 2, pp. 8–11, 2008.

[7] J. Oksanen, F. G. Blanchet, M. Friendly, R. Kindt, P. Legendre, D. McGlinn, P. R. Minchin, R. B. O'Hara, G. L. Simpson, P. Solymos, M. H. H. Stevens, E. Szoecs, and H. Wagner, *vegan: Community Ecology Package*, 2017. R package version 2.4-4.

[8] M. A. Rodríguez-Gironés and L. Santamaría, "A new algorithm to calculate the nestedness temperature of presence–absence matrices," *Journal of Biogeography*, vol. 33, no. 5, pp. 924–935, 2006.

[9] C. Robertson, "Flowers and insects lists of visitors of four hundred and fifty three flowers," 1929.

[10] *rARPACK: Solvers for Large Scale Eigenvalue and SVD Problems.* Available at: https://CRAN.R-project.org/package=rARPACK.

[11] E. Burgos, H. Ceva, R. P. Perazzo, M. Devoto, D. Medan, M. Zimmermann, and A. M. Delbue, "Why nestedness in mutualistic networks?," *Journal of theoretical biology*, vol. 249, no. 2, pp. 307–313, 2007.

[12] C. Payrató-Borràs, L. Hernández, and Y. Moreno, "Breaking the spell of nestedness: The entropic origin of nestedness in mutualistic systems," *Physical Review X*, vol. 9, no. 3, p. 031024, 2019.

[13] E. Jones, T. Oliphant, P. Peterson, *et al.*, "SciPy: Open source scientific tools for Python," 2001–.

[14] S. Seabold and J. Perktold, "Statsmodels: Econometric and statistical modeling with python," in *9th Python in Science Conference*, 2010.
